# Important situations that capture moral distress in paediatric oncology

**DOI:** 10.1186/s12910-020-0447-x

**Published:** 2020-01-13

**Authors:** Margareta af Sandeberg, Cecilia Bartholdson, Pernilla Pergert

**Affiliations:** 1 Department of Women’s and Children’s Health, Childhood Cancer Research Unit, Karolinska Institutet, Tomtebodavägen 18A, SE-171 77 Stockholm, Sweden; 2 Paediatric Haematology and Oncology, Children’s and Women’s Health Care, Karolinska University Hospital, Stockholm, Sweden; 3 Paediatric Neurology and Muscular Skeletal Disorders and Homecare, Children’s and Women’s Health Care, Karolinska University Hospital, Stockholm, Sweden

**Keywords:** Moral distress, Paediatric oncology, Healthcare professionals, Registered nurses, Medical doctors, Nursing assistants, Hospital settings

## Abstract

**Background:**

The paediatric Moral Distress Scale-Revised (MDS-R) was previously translated and adapted to Swedish paediatric oncology. Cognitive interviews revealed five not captured situations among the 21 items, resulting in five added items: 22) Lack of time for conversations with patients/families, 23) Parents’ unrealistic expectations, 24) Not to talk about death with a dying child, 25) To perform painful procedures, 26) To decide on treatment/care when uncertain. The aim was to explore experiences of moral distress in the five added situations in the Swedish paediatric MDS-R, among healthcare professionals (HCPs) in paediatric oncology.

**Methods:**

In this national cross-sectional survey, the Swedish paediatric MDS-R, including five added items, were used. Descriptive statistics, non-parametric analysis of differences between professions and a MDS-R score for each item were calculated. Internal consistency was tested using Cronbach’s alpha and inter-item correlation test. HCPs (*n* = 278) at all six Swedish paediatric oncology centres participated (> 89%). The Regional Ethical Review Board had no objections. Consent was assumed when the survey was returned.

**Results:**

Nursing assistants (NAs) reported higher intensity and lower frequency on all added items; registered nurses (RNs) reported a higher frequency on item 22–25; medical doctors (MDs) reported higher MDS-R score on item 26. On item 22, intensity was moderate for RNs and MDs and high for NAs, and frequency was high among all. Item 22, had the second highest MDS-R score of all 26 for all professional groups. On item 23, the level of disturbance was low but it occurred often. The 26-item version showed good internal consistency for the overall sample and for all professional groups. However, item 22 and 24 could be viewed as redundant to two of the original 21.

**Conclusion:**

In accordance with other studies, the intensity was higher than the frequency, however, the frequency of the added items was higher than of the original items. In line with previous research, item 22 and 23 are important elements of moral distress. RNs experience the situations more often while NAs find them more disturbing. The results indicate that the added items are important in capturing moral distress in paediatric oncology.

## Background

Moral distress is an experience shared by all healthcare professionals (HCPs), although different professions have different perspectives on it and experiences of it [1]. In previous studies, moral distress has been shown to be related to burnout, low job-satisfaction and high turnover of HCPs [2, 3]. According to an analysis of the theory of moral distress, the main problem is, apart from the negative effects on HCPs, the impact on the quality of patient care [4].

Moral distress has been defined as a consequence when someone knows what is ethically right but for different reasons cannot act accordingly [5, 6]. According to O’Donnell et al. [7] moral distress also includes a consequence of a moral dilemma, that is a conflict of values when one has to take a decision when not knowing what is ethically right. Previous studies on differences in levels of moral distress between nurses and medical doctors (MDs) show contradictory results [8, 9]. A recent study found no significant differences between the total moral distress scores of nurses and MDs [10]. However, in a multi-professional study in Swedish paediatric oncology, registered nurses (RNs) rated significantly higher frequencies and higher total moral distress than MDs and nursing assistants (NAs) [11]. By understanding the causes of moral distress, interventions can be planned to help HCPs handle moral distress [1].

The Moral Distress Scale (MDS) was developed by Corley et al. to measure moral distress among nurses in hospital settings [12]. When revising the MDS (MDS-R) Hamric et al. [13] shortened it to include 21 items describing clinical situations found to generate moral distress among HCPs, and paediatric versions were developed for nurses, MDs and others.

When the paediatric MDS-R was translated and culturally adapted to Swedish paediatric oncology in 2014–2015, the target population was HCPs (RNs, MDs and NAs) in hospital settings [14]. The aim was to keep the original content as much as possible to be able to compare results across studies. Furthermore, the aim was to make it clinically relevant to the paediatric oncology context. This is in contrast to the Measure of Moral Distress for Health Care Professionals (MMD-HP) developed by Epstein et al. [15] who was aiming for a more generic version. Epstein et al. increased the number of items at team-level (such as poor communication and interaction) and system level (for example excessive requirements of documentation). In the process of cognitive debriefing of the Swedish paediatric MDS-R, two focus group interviews and 16 individual cognitive interviews were performed in accordance with Collins [16] with HCPs, including RNs, MDs and NAs, in paediatric oncology at University Hospitals [14]. Field notes were taken and discussed in a review group (*n* = 7) with expertise in paediatric oncology, ethics, translation and questionnaire design. The situations described in the 21 items in the Swedish translation of the original MDS-R were found to be relevant [14]. However, some adjustments were necessary, for example, the indicated frequency was found to influence how the respondents indicated the level of disturbance (intensity), and thus the intensity was placed before the frequency in the Swedish MDS-R. Furthermore, situations not captured among the original 21 items were identified resulting in five added items describing situations that all HCPs in close patient care encounter including: 22) Lack of time for conversations with patients/families, 23) Parents’ unrealistic expectations, 24) Not to talk about death with a dying child, 25) To perform painful procedures, 26) To decide on treatment/care when uncertain. Thus, the decision to add these items was based on empirical data rather than theory.

The aim of the present study was to explore experiences of moral distress in five specific situations in the Swedish paediatric MDS-R among HCPs in Swedish paediatric oncology. The aim is also to determine if the items should be permanently included when measuring moral distress in this context.

*Research questions:*

- How frequent are the situations described and are there differences between professions?
- How much do the situations described disturb the HCPs and are there differences between professions?
- What are the levels of moral distress in the situations described and are there differences between professions?
- How are the five items ranked among the 26 items and are there differences between professions?

## Methods

### Study design

This study is part of a larger national cross-sectional survey in childhood cancer care, including all six paediatric oncology centres situated at a University Hospital in each of the six national healthcare regions (Gothenburg, Linköping, Lund, Stockholm, Umeå, and Uppsala) in Sweden. This part of the study focuses on the five items in the Swedish paediatric MDS-R that were not included in the original MDS-R, by Hamric et al. [13].

### Data collection

Managers of the paediatric oncology centres were informed about the study and all the six centres participated. The data collection was conducted during joint unit meetings arranged by the paediatric oncology centres and those unable to attend were, in collaboration with local coordinators, offered the opportunity to answer the questionnaire afterwards. Written and oral information, about confidentiality and final reporting of results exclusively on a group level, was provided to all participants. It was also emphasized that participation was voluntary. A total of 309 HCPs (167 RNs, 70 MDs and 72 NAs) were invited to participate. The data collection included demographic data (gender, profession, years in paediatrics, and paediatric oncology centre) and the Swedish paediatric MDS-R. The Swedish paediatric MDS-R consists of 21 + 5 items describing situations that could generate moral distress. HCPs are asked, without any specified recall time, to rate, on a 5-point (0–4) Likert scale, how much the situation described would disturb them (intensity, not at all-very negatively) and how often they experienced this situation (frequency, never-very often).

### Data analysis

Focusing on the added items, the analysis included descriptive statistics: frequency, mean, median, standard deviation (SD) and range. A moral distress score (henceforth MDS-R score) for each item was calculated as a composite score, i.e. intensity and frequency was multiplied (range 0–16) with higher scores suggesting higher levels of moral distress. The MDS-R score was used to enable the comparison with other studies. Less than 10% missing responses were considered acceptable [17]. The classification of low, moderated and high scores were based on the percentiles of the means of the 21 items of the Swedish paediatric MDS-R in paediatric oncology (Table 1).

**Table 1:** The classification of low, moderate and high scores of intensity and frequency

	Low (25% percentile)	Moderate	High (75% percentile)
Intensity	< 3.29	3.29–3.50	> 3.50
Frequency	< 0.62	0.62–1.29	> 1.29

The differences in mean values of intensity, frequency, and MDS-R score between professions were tested using the Mann-Whitney U test. *P*-values < 0.05 were considered statistically significant. The internal consistency of both the 21-item and the 26-item versions of the Swedish paediatric MDS-R were calculated using Cronbach’s alpha and inter-item correlations. Cronbach’s alpha ≥0.80 and inter-item correlation ranging between 0.15–0.50 are suggested to show good internal consistency [18]. All statistical calculations were conducted using Statistical Package for Social Sciences (SPSS) version 22.0 (IBM SPSS Statistics, Inc., Chicago, IL).

## Results

### Participants

Of 278 participants 83% were female and 17% male. The total response rate (RR) was > 89%, and the median at the six centres was 91% (range 78–98%). The distribution of HCPs was 56% RNs (RR 83,3%), 20% MDs (RR 89,5%), and 24% NAs (RR 95,68%). In Sweden NAs have an upper secondary education, thus not licensed and not university trained as RNs. Missing items among the five items were less than 3 % (range 0.7–2.9).

### Levels of moral distress related to the added items

On the five items together, RNs reported significantly higher frequency than the other two professions while NAs reported significantly higher moral distress intensity and lower frequency than RNs and MDs (Table 2).

**Table 2:** Moral distress intensity and frequency, in the entire group and by profession, 5 items together

	All	RNs	MDs	NAs	RNs vs. MDs	RNs vs. NAs	MDs vs. NAs
Category	n = 278	*n* = 157	*n* = 55	*n* = 66	*p*-value		
Intensity:							
Mean (SD)	3.10 (0.60)	3.09 (0.57)	2.95 (0.55)	3.25 (0.66)	0.093	0.029	0.002
Median (Range)	3.20 (0.60–4.00)	3.20 (0.80–4.00)	3.00 (1.80–4.00)	3.40 (0.60–4.00)			
Frequency:							
Mean (SD)	1.46 (0.63)	1.60 (0.61)	1.40 (0.47)	1.19 (0.68)	0.022	<0.001	0.044
Median (Range)	1.40 (0.00–3.60)	1.60 (0.00–3.60)	1.40 (0.60–2.60)	1.00 (0.00–3.00)			

NAs also reported higher moral distress intensity than the other professions on all the five situations. All situations, except item 26, were reported to have occurred more often by RNs than the other professions (Table 3).

**Table 3:** Intensity, frequency, and MDS-R scores, by all and by profession, on the 5 items

	All	RNs	MDs	NAs
22. To not have time to have conversations with patients and families in a way that you think these should be carried out.				
Intensity, Mean (SD)	3.45 (0.699)	3.41 (0.699)	3.45 (0.715)	3.52 (0.687)
Intensity, Median (Range)	4.00 (1–4)	4.00 (1–4)	4.00 (1–4)	4.00 (1–4)
Frequency, Mean (SD)	1.86 (1.015)	2.08 (0.984)	1.75 (0.907)	1.42 (1.036)
Frequency, Median (Range)	2.00 (0–4)	2.00 (0–4)	2.00 (0–4)	1.00 (0–4)
MDS-R score	6.45	7.17	6.04	5.11
23. To provide care despite that parents have unrealistic expectations of healthcare.				
Intensity, Mean (SD)	2.55 (0.993)	2.53 (1.015)	2.29 (0.916)	2.82 (0.950)
Intensity, Median (Range)	2.00 (0–4)	2.00 (0–4)	2.00 (1–4)	3.00 (1–4)
Frequency, Mean (SD)	1.91 (9.989)	1.99 (1.016)	1.95 (0.911)	1.68 (0.964)
Frequency, Median (Range)	2.00 (0–4)	2.00 (0–4)	2.00 (0–4)	2.00 (0–4)
MDS-R score	5.01	5.17	4.67	4.89
24. To not talk about death with a dying child, despite that you think it is necessary.				
Intensity, Mean (SD)	3.46 (0.766)	3.41 (0.763)	3.47 (0.742)	3.56 (0.794)
Intensity, Median (Range)	4.00 (0–4)	4.00 (0–4)	4.00 (1–4)	4.00 (0–4)
Frequency, Mean (SD)	1.02 (0.835)	1.09 (0.881)	0.93 (0.716)	0.92 (0.809)
Frequency, Median (Range)	1.00 (0–4)	1.00 (0–4)	1.00 (0–3)	1.00 (0–3)
MDS-R score	3.56	3.84	3.11	3.33
25. To perform painful/unpleasant procedures on school-age children who resist such treatment.				
Intensity, Mean (SD)	2.93 (0.994)	2.93 (1.017)	2.76 (0.889)	3.10 (1.011)
Intensity, Median (Range)	3.00 (0–4)	3.00 (0–4)	3.00 (1–4)	3.00 (1–4)
Frequency, Mean (SD)	1.58 (1.065)	1.78 (1.106)	1.17 (0.746)	1.42 (1.081)
Frequency, Median (Range)	1.00 (0–4)	2.00 (0–4)	1.00 (0–3)	1.00 (0–4)
MDS-R score	4.29	4.86	2.94	4.13
26. To decide on care/treatment when you are uncertain about what is right.				
Intensity, Mean (SD)	3.08 (0.972)	3.16 (0.889)	2.76 (0.942)	3.18 (1.138)
Intensity, Median (Range)	3.00 (0–4)	3.00 (0–4)	3.00 (1–4)	4.00 (0–4)
Frequency, Mean (SD)	0.92 (0.752)	0.95 (0.701)	1.20 (0.704)	0.58 (0.801)
Frequency, Median (Range)	1.00 (0–3)	1.00 (0–3)	1.00 (0–3)	0.00 (0–3)
MDS-R score	2.72	2.90	3.15	1.92

In the situation described in item 22 (Lack of time for conversations with patients/families), the overall moral distress intensity was moderate and the frequency scores was high. RNs rated higher MDS-R score on item 22 than the other two professional groups (Table 3). In the situation described in item 23 (Parents unrealistic expectations), the moral distress intensity was low but it occurred often (high frequency). RNs rated higher MDS-R scores on this item [19] than did the two other professional groups (Table 3). In the situation described in item 24 (Not talk about death with a dying child), both the moral distress intensity and frequency were moderate (Table 3). In the situation described in item 25 (Perform painful procedures), the moral distress intensity score was low and the frequency score was high in the entire group. The MDS-R score on this item [20] was higher for RNs than for NAs, because RNs encountered the situation more often. The MDS-R scores of RNs and NAs were higher than those of MDs (Table 3). In the situation described in item 26 (Decide when uncertain), the moral distress intensity of this situation was low, and slightly lower for MDs. The frequency of this situation [21] was moderate, and slightly higher for MDs. The MDs score was higher than those of both RNs and NAs (Table 3).

### MDS-R scores and ranking

**Fig. 1:**
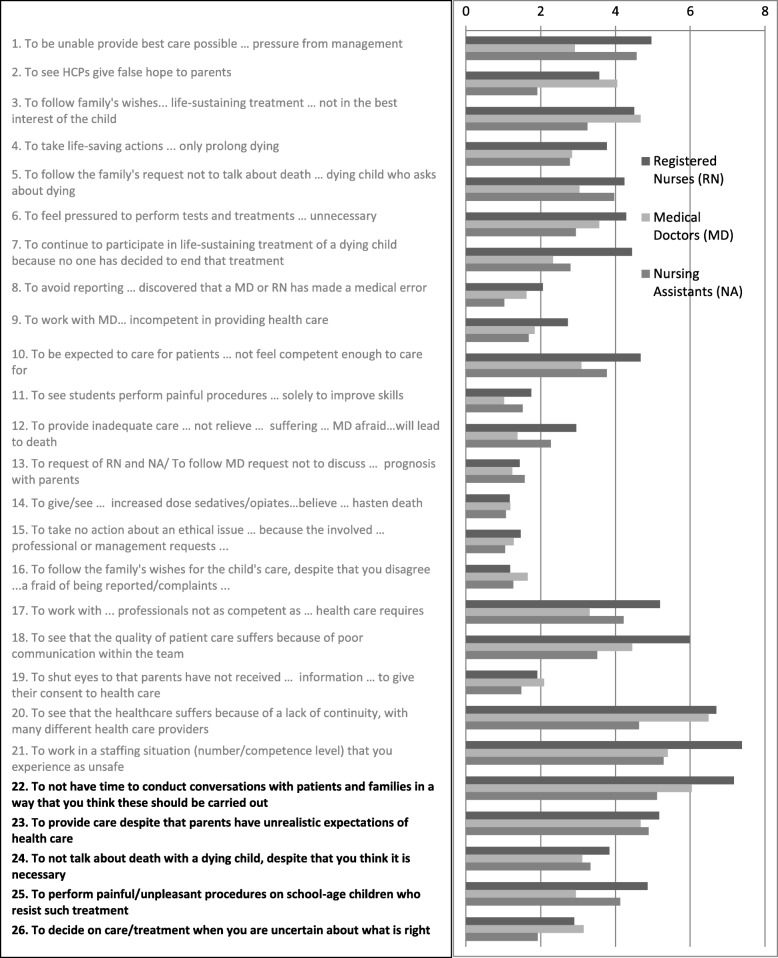
Mean MDS-R scores per professional group in the Swedish paediatric MDS-R, 26 items

In the results from all 26 items in the Swedish paediatric MDS-R (the original 21 items and the added five items), the RNs rated the highest MDS-R scores in 19 of the 26 items compared to the other professional groups (Fig. 1). The MDs rated six of the 26 items, including one of the five, higher than RNs and NAs. Of these, three items generated levels of MDS-R scores that were similar to those of the RNs, but clearly higher than those of the NAs. These items concerned others giving false hope to parents, giving treatment not in the best interests of the child as well as deciding when uncertain. The NAs rated one single item, concerning not discussing the prognosis with parents, as slightly higher than the others.

Regarding the situation described in item 22 (Lack of time for conversations with patients/families) the MDS-R score of this situation was the second highest of the 26 items in the entire group and also for each of the professional groups (Table 4).

**Table 4:** MDS-R scores and ranking of 5 items among 26 items, by all and by profession

	All		RNs		MDs		NAs	
Items	MDS-R score	Rank of 26	MDS-R score	Rank of 26	MDS-R score	Rank of 26	MDS-R score	Rank of 26
22 To not have time to conduct conversations with patients and families in a way that you think these should be carried out.	6.45	2	7.17	2	6.04	2	5.11	2
23 To provide care despite that parents have unrealistic expectations of healthcare.	5.01	5	5.17	6	4.67	4	4.89	3
24 To not talk about death with a dying child, despite that you think it is necessary.	3.56	14	3.84	14	3.11	11	3.33	11
25 To perform painful/unpleasant procedures on school-age children who resist such treatment.	4.29	8	4.86	8	2.94	14	4.13	7
26 To decide on care/treatment when you are uncertain about what is right.	2.72	17	2.90	18	3.15	10	1.92	17

The situation described in item 23 (Parents unrealistic expectations) scored as number five of the 26 items, by all HCPs. The MDS-R score on item 23, by each professional group, was among the top six on the ranking list (Table 4). Even though RNs rated a higher MDS-R score than the other two professional groups, this item [19] scored lower on the ranking list for RNs, than for NAs and MDs (Table 4). The situation described in item 24 (Not talking about death with a dying child) scored as number 14 in the entire group and for RNs, and as number 11 for both MDs and NAs (Table 4). On the situation described in item 25 (Perform painful procedures), the MDS-R score of RNs and NAs were higher than for MDs. This item [20] scored higher, on the 26-item ranking list, for RNs and NAs than for MDs. The situation described in item 26 (Decide when uncertain) scored as number 17 on the 26-item ranking list, in the entire group. This item [21] scored as number 18 by RNs, similar to that of NAs. The MDS-R score of MDs was higher than those of both RNs and NAs, scoring as number 10 of the ranking list for MDs (Table 4).

### Reliability

Both the original 21-item and the 26-item versions showed good internal consistency for the overall sample and for all professional groups. Cronbach’s alpha was slightly higher for the 26-item than for the original 21-item version (Table 5).

**Table 5:** Reliability testing of the Swedish paediatric MDS-R with and without the added items

	All	RNs	MDs	NAs
Version	Cronbach’s alpha			
21 items	0.882	0.878	0.807	0.909
26 items	0.905	0.903	0.822	0.928

The inter-item correlation test suggested that two of the five added items were redundant with two of the 21-items. The inter-item correlation between item 22 (Lack of time for conversations with patients/families) and item 21 (To work in an unsafe staffing situation) was 0.55. The inter-item correlation between item 24 (Not talking about death with a dying child) and item 5 (To follow the family’s request not to talk about death … dying child) was 0.55.

## Discussion

This national cross-sectional survey at all paediatric oncology centres in Sweden focuses on five situations (items) added to the Swedish paediatric MDS-R. The five items were added in a previous study because participants in cognitive interviews considered them to be important [14]. The appropriateness of adding items to validated instruments could be questioned as it, for example, makes it difficult to compare the total MDS-R scores with other studies, and for that reason the items were initially not included in the previously published results from this national study and in the analysis of the Swedish total MDS-R scores [11]. However, to maintain the content validity also validated instruments need to be adapted both to the specific context and to changes over time [22]. Furthermore, adding items of relevance to the specific context of paediatric oncology improved the relevance of the instrument and possibly also the response rate. In accordance with other studies on moral distress in paediatric settings, the overall moral distress intensity was comparatively higher than the frequency [9, 23]. Also in the results from the 21 items of the paediatric MDS-R in this Swedish study, the situations were experienced as disturbing (3.3), but not that common (1.0) [11]. In accordance with the results of the cognitive interviews, the five added situations were more common, because the frequency of the five items together was higher (1.46) than of the 21 items. Thus, we would argue that the five items should be permanently included when measuring moral distress in the paediatric oncology context.

As described in previous research, “Lack of time for conversations with patients/families” was, by each of the professional groups, considered to be one of the most morally distressing situations. However, the results of the inter-item correlation test indicate that this added item could be viewed as redundant to the item “To work in an unsafe staffing situation”. This is in accordance to Atabay et al. [﻿24﻿], who presents lack of time and resources as one dimension of moral distress. The time aspect was also highlighted when nurses working part-time experienced more moral distress than nurses working full time [25]. A constant pressure of time, a lack of staff, and too many patients, leads to constant ethical challenges and a feeling of not being able to influence the situation [﻿24﻿]. It is puzzling that these results are from upper middle- and high-income countries with resources in equipment and staff that HCPs in low-income countries could only dream of. One possible reason is that standard of care varies depending on resources and that it is morally distressing not being able to provide care at the same level that you have been used to [﻿19﻿]. However, even if there is a difference in the experience of moral distress depending on context, lack of resources to relieve patients’ suffering has also been found to lead to moral distress in a low-income country [26].

“Parents’ unrealistic expectations” were, in accordance with other studies, scored highly by all HCPs. Even though the moral distress intensity was low, the situation occurred often and thus there is a risk of the crescendo effect when HCPs repeatedly experience morally distressing situations, with increased moral distress over time, due to a build-up of moral residue [﻿20﻿]. However, the low intensity could also be explained by a learning effect due to the high frequency [﻿21﻿]. Differences in parent and HCPs’ views concerning prognosis and goals have been suggested as being common in paediatric cancer care [27]. Furthermore, in previous studies HCPs have reported that unrealistic parental expectations hinder the communication with children having life-threatening conditions [28, 29]. Parents’ prognostic understanding and attitudes have been identified as being the most common barriers to discussing advanced care planning with the children [28]. In a study of paediatric palliative care, HCPs perceived parent’s unrealistic expectations as complicating the shared decision-making with patients and parents [30]. Furthermore, the unrealistic expectations of parents could be related to them being misinformed and one of three suggested dimensions of moral distress include misinformed patients, which here can be attributed to parents [﻿24﻿].

One of the five added items was item 24 “To not talk about death with a dying child” [14]. The decision to include that item can be questioned, as it could be argued that this situation was already included in the original version of MDS-R, also supported by the results of the inter-item correlation test. The difference is that the original item includes a request from the family, i.e. two questions for the respondents to consider: not talking and following family’s request [14]. Our results indicate that not talking about death with a dying child is slightly more morally distressing when requested by the family (Fig. 1), especially for RNs and NAs. However, two questions in one, also called a double-barrelled question, has been described as a bias in question design [31]. Furthermore, in the translation process of the original MDS-R, cognitive interviews revealed that not talking about death, for whatever reason, is a very distressing situation [14]. Furthermore, other researchers have described the emotional challenge for HCPs when parents decide to limit the information to the child as well as when the child refuses to talk about death to spare the parents [32]. Also, the nurses in a study by Hendricks-Ferguson et al. [33] described the inability to respond to unexpected emotional comments from the dying children as very distressing, causing guilt and regret. Another reason for not being able to reach out to the child is language barriers [34, 35]. The results of a review of 65 studies in paediatric end-of-life care showed that both parents and HCPs have a tendency to avoid discussions about death with children [36]. In one study, children expressed a hypothetical desire of being informed if the treatment were not to be successful [37].

On the item about “Performing painful procedures on children who resist”, RNs scored higher frequency than NAs and MDs. This is not surprising because, in Sweden painful procedures, on children that are not sedated, are primarily performed by RNs. In the literature, procedures performed against patient’s wishes are most often discussed together with unnecessary treatment [38, 39]. In the original MDS-R, there are items about unnecessary treatment, but there was no item capturing disregard for patient wishes [14] which has been identified as a clinical situation causing moral distress [13]. Furthermore, performing painful procedures has emerged as an important ethical issue in paediatric cancer care [39].

“To decide when uncertain” is a situation consistent with the second definition of moral distress, which includes a moral dilemma, and it has been assumed to match physicians’ experiences more closely [6]. In accordance with this assumption, this situation caused the MDs more moral distress, related to higher frequency of this situation, than the other professions in this study. Even though it was scored by the whole group as number 17 among the 26 items, the overall moderate frequency indicate that the item does not fully capture these situations. One reason for this could be that the item is too generally designed, and maybe it would have been better to describe more concrete situations with moral uncertainty in decision-making. In a previous study, paediatric oncologists describe that they on a daily basis face difficult decisions that they prefer not to take by themselves [40]. The increased survival rate and the never-ending availability of non-standard treatment options in paediatric oncology has made the decisions even more difficult [3]. Furthermore, the practice of shared decision-making with the patients or proxy has been suggested to further increase the moral distress among physicians [41].

In contrast to the development of MMD-HP, that used data from several diverse settings, the decision to add the five items was entirely based on data from paediatric oncology, to better capture moral distress in that setting [14, 15]. When comparing the five added items in the Swedish paediatric MDS-R to the MMD-HP by Epstein et al. [15] there are some similarities in four of them. Item 22 (Lack of time for conversations with patients/families) is partly covered in the MMD-HP with an item concerning compromised patient care due to excessive documentation requirements. Both items focus indirectly on missed care due to lack of time, however, the MMD-HP gives one specific reason for the lack of time. Item 23 (Parents unrealistic expectations) is similar to an item about patients who have unclear or inconsistent treatment plans or who lack goals of care. In paediatric care, however, this item would be more applicable to parents. Furthermore, in MMD-HP the item “to follow the family’s request not to talk about death with a dying child” has been removed and the situation has possibly been included in the item “Follow a physician’s or family member’s request not to discuss the patient’s prognosis with the patient/family.” In item 24 (Not to talk about death with a dying child) in the present study, the reason for not talking was excluded in accordance to the aim to avoid double-barrelled questions. However, adding several reasons might have the same effect as the item is no longer about the individual reasons [42]. Item 25 (Painful procedures on children who resist) in the present study captures a situation that is part of everyday care in paediatrics where HCPs are not always able to practice care as they think it should be practiced. Maybe the first part of the item in MMD-HP “Participate in care that causes unnecessary suffering …” is similar, but it is questionable if it captures the feeling of being the cause of the suffering.

Strengths of this study are that it is national and multi-professional as it includes HCPs at all paediatric oncology centres. Furthermore, despite the extreme work load and the emphasis on the voluntary participation, the response rate was high which could possibly be explained by the relevance of the questionnaire but also that data collection was mainly performed during meetings when HCPs had time set aside to complete the questionnaire. A possible limitation of this study is that the statistical analyses do not take into account possible dependencies between participants of the same centre. A non-parametric statistical test was preferred rather than applying a parametric regression model to adjust for possible centre effects. Most studies, see for example Hamric et al. [8] and Whitehead et al. [1], only report the MDS-R scores, while in the present study both the MDS-R scores as well as the moral distress intensities and frequencies are reported. The calculation of the MDS-R score could be questioned, as the experience of the intensity of the moral burden is always inseparably influenced by its frequency, but it enables comparison with other studies. In interpreting the results it is important to consider both the possible crescendo effect and the learning effect [﻿20﻿, ﻿21﻿]. Furthermore, in this study Cronbach’s Alpha has been used to test reliability. In future research, the psychometric properties could be further evaluated.

## Conclusions

RNs experience the highest moral distress in four of the five added items compared to the other professions. MDs experience higher moral distress in the situation about deciding when uncertain, because they experience the situation more often. Furthermore, it is clear that RNs experience the situations more often than NAs do, while NAs find them more disturbing. The results indicate that the added situations occur often and are important in capturing moral distress in paediatric oncology. Thus, four of the added items should be included in the original MDS-R, and the fifth reformulated item about not talking about death with a dying child should replace the original item when measuring moral distress in the context of paediatric oncology and similar hospital settings. Further refinement of the MMD-HP could include to develop a paediatric version that captures the situations presented in the present study.

## Data Availability

Due to respect for the participant’s anonymity the data collected and analysed in the current study are not publicly available but may be available from the corresponding author on reasonable request.

## Abbreviations

HCP: Healthcare professional;
MD: Medical doctor
MDS: Moral distress scale
MDS-R: Moral distress scale-revised
NA: Nursing assistant
RN: Registered nurse
RR: Response rate
SPSS: Statistical Package for Social Sciences
